# High Expression of *SOX2* Is Associated with Poor Prognosis in Patients with Salivary Gland Adenoid Cystic Carcinoma

**DOI:** 10.3390/ijms15058393

**Published:** 2014-05-13

**Authors:** Wei Dai, Xuexin Tan, Changfu Sun, Qing Zhou

**Affiliations:** Department of Oromaxillofacial-Head and Neck Surgery & Department of Oral and Maxillofacial Surgery, School of Stomatology, China Medical University, Shenyang 110002, China; E-Mails: daicmu@163.com (W.D.); xuexintan@sohu.com(X.T.); suncfu@yeah.net (C.S.)

**Keywords:** sex determining region *Y-BOX2*, adenoid cystic carcinoma, prognosis, biomarker

## Abstract

Sex determining region *Y-BOX2* (*SOX2*), one of the key members of the *SOX* family, is a transcription factor that is involved in the maintenance of embryonic stem cell pluripotency and in multiple developmental processes. Recent studies have shown that *SOX2* is aberrantly expressed in several types of tumors. The present study aimed to investigate the clinicopathological and prognostic significance of *SOX2* in adenoid cystic carcinoma (ACC) of salivary gland. In this study, the expression of *SOX2* in ACC tissues and matched adjacent non-cancerous tissues was measured by immunohistochemistry, western blot, and quantitative polymerase chain reaction. High *SOX2* expression occurred in approximately 62.6% of primary ACC. In addition, high expression of *SOX2* was significantly associated with T classification (*p* = 0.003) and distant metastasis (*p* = 0.002). The 5-year overall survival (OS) and disease-free survival (DFS) in patients with high *SOX2* expression is poorer than those with low *SOX2* expression. When adjusted by multivariate analysis, high *SOX2* expression, together with distant metastasis, was an independent prognostic factor. The findings of the present study provide evidence that *SOX2* represents a potential novel prognostic biomarker for ACC patients.

## 1. Introduction

Adenoid cystic carcinomas (ACC) are rare variants of adenocarcinoma that most often arise in the salivary glands, and account for 25% of malignant tumors in the major salivary glands, and 50% in the minor glands [1,2]. Although ACC tends to grow slowly, this neoplasm has a poor prognosis, owing to its diffuse invasion, high incidence of local recurrence and distant metastasis [3]. To date, the molecular mechanisms for the aggressive invasiveness remain unclear. Therefore, it will be of great clinical value to identify the key regulatory molecules associated with the development and progression of ACC for early detection and prognosis and for the development of novel interventions.

Recent studies demonstrated that cancer cells with stem/progenitor cell properties exhibit enhanced invasive properties, supporting the concept that cancer stem cells (CSCs) may play an important role in tumor development [4–6]. CSCs have the ability to apoptosis-resistant, self-renew and differentiate into multiple cell lineages, thereby generating tumor heterogeneity. To date, the possible existence of CSCs has been identified in leukemia [7], breast cancer [8,9], gastric cancer [10], cervical cancer [11], and other malignancies [12,13]. The presence of CSCs in ACC is likely one of the main causes of tumor progression, metastasis, recurrence, and resistance to therapy [14,15].

The sex determining region *Y-BOX* (*SOX*) genes encode a family of high-mobility groups that are a family of transcriptional factors and have emerged as potent modulators involved in orchestrating embryonic development and cell fate, organogenesis, stem cells maintenance, and cancerogenesis in multiple processes [16]. So far, 20 different *SOX* genes have been discovered in mice and humans [17]. *SOX2*, one of the key members of the *SOX* family gene, is a transcription factor that is involved in the maintenance of embryonic stem cell pluripotency and in multiple developmental processes. Recent studies have shown that *SOX2* is aberrantly expressed in several types of tumors, including breast, lung, prostate, ovarian, gastric cancer, and melanomas [18–23]. Moreover, *SOX2* expression pattern and the correlation with histopathological status and clinical outcome are highly variable among tumors, suggesting distinct roles of *SOX2* in individual tumors. However, to our knowledge, little is known about the expression and role of *SOX2* in ACC. In this study, we used immunohistochemistry (IHC), western blot, and quantitative polymerase chain reaction (qPCR) to evaluate the expression of *SOX2* in 131 ACC tissues specimens. Moreover, this study investigated the relationship between *SOX2* expression and clinical and pathological features of ACC patients, and assessed its potential prognostic value in patients with ACC.

## 2. Results

### 2.1. Expression of Sex Determining Region Y-BOX2 (SOX2) in Adenoid Cystic Carcinomas (ACC) Tissue and Non-Cancerous Tissue

**Figure 1.**
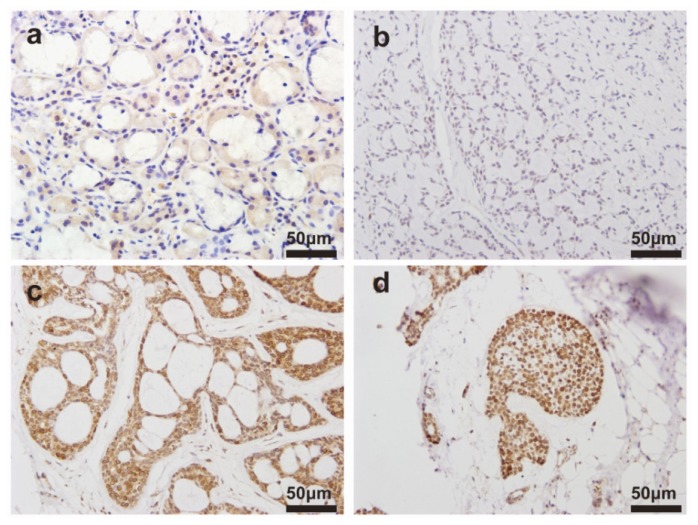
Immunohistochemistry (IHC) analysis of representative expression patterns of sex determining region *Y-BOX2* (*SOX2*) in the adenoid cystic carcinomas (ACC) tissue and adjacent non-cancerous tissues: (**a**) adjacent non-cancerous tissue; and (**b**–**d**) ACC tissues. Positive *SOX2* staining in ACC tissues appeared as brown particles which were mainly localized within the nucleus of epithelial cells of glands. Original magnification: all 400×. Scale bar, 50 μm.

**Figure 2.**
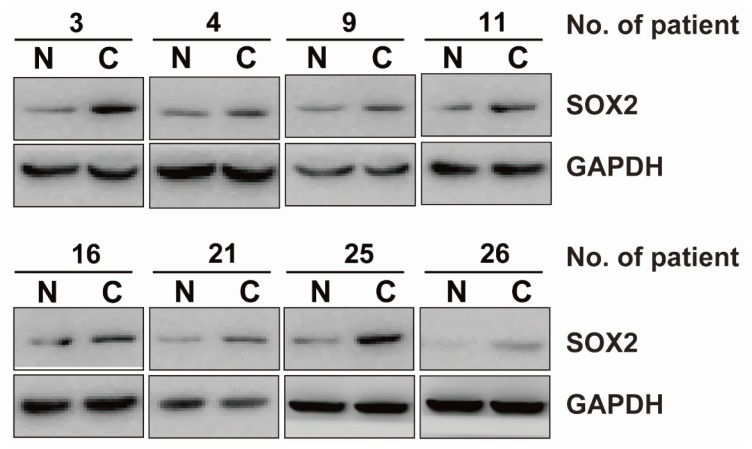
Western blot analysis on the expression of *SOX2* in ACC tissue and adjacent non-cancerous tissues. Lysates of ACC tissues (C) and matched adjacent non-cancerous tissues (N) were analyzed with western blot. The representative eight pairs are shown.

**Figure 3.**
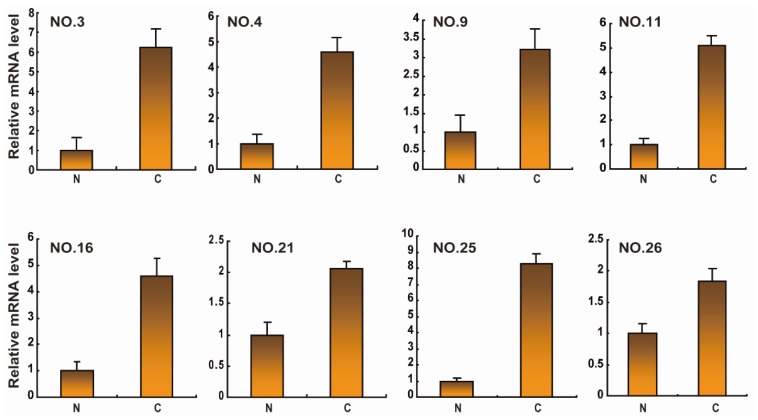
The mRNA expression of *SOX2* in ACC. Evaluation of the expression of the indicated mRNA using quantitative polymerase chain reaction (qPCR). Lysates of 30 ACC cancer tissues (C) and matched adjacent non-cancerous tissues (N) pairs were analyzed using real-time polymerase chain reaction (PCR). The representative eight pairs are shown.

The clinical characteristics of ACC patients were summarized in Table 1. We observed the expression levels and subcellular localization of *SOX2* protein in ACC tissue and adjacent non-cancerous tissue using immunohistochemical staining. Specific *SOX2* protein staining was mainly detected in the nucleus of ACC cells (Figure 1). Increased expression of *SOX2* was observed in ACC tissue compared to matched adjacent non-cancerous tissues. The frequency of high expression of *SOX2* in ACC tissue was 62.6% (82/131). Western blotting analysis showed that SOX2 protein was upregulated in ACC tissues compared with matched adjacent non-cancerous tissues (Figure 2). *SOX2* expression was further analyzed using quantitative polymerase chain reaction (qPCR) analysis. In qPCR analysis, the amplification efficiency of *SOX2* and β-actin were 95% and 98%, respectively (Figure S1). Moreover, the gene expressions of β-actin showed no difference in diverse ACC tissues and non-cancerous tissues. Most malignant ACC tissues demonstrated increased expression of *SOX2* when compared with non-cancerous tissue (Figure 3).

### 2.2. Association between the Expression of SOX2 and Clinicopathological Features

In order to further explore the role of *SOX2* in the development of ACC, the relationship between *SOX2* expression and clinicopathological parameters was analyzed. As shown in Table 1, *SOX2* expression is noted in each stage of the tumor, while high *SOX2* expression is more frequently noted in the ACC tissues with higher T classification and distant metastasis. There was no significant association between *SOX2* expression and age, gender, histological type, and nerve invasion.

### 2.3. SOX2 Expression and Prognostic Relevance

**Figure 4.**
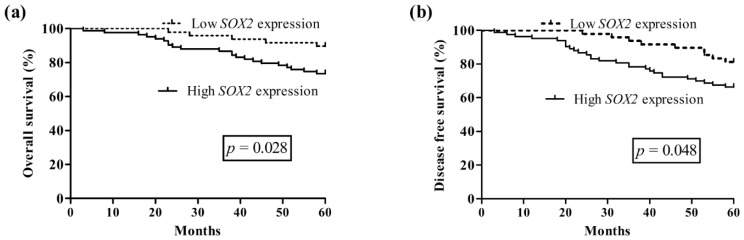
Kaplan-Meier survival curves for overall survival and disease-free survival for *SOX2*: (**a**) overall survival; and (**b**) disease-free survival.

Follow-up information was available on 131 ACC patients for periods ranging from 3 to 62 months (mean, 38.6 months). Kaplan-Meier survival analysis was adopted to evaluate *SOX2* expression and survival time of patients with ACC. The survival curves were stratified according to *SOX2* expression. As shown in the log-rank tests in Figure 4, overall survival (OS) and disease-free survival (DFS) of ACC patients with high *SOX2* expression were lower than that of patients with low *SOX2* expression (OS, *p* = 0.028; DFS, *p* = 0.048, respectively) (Figure 4). It is evident that *SOX2* may be a significant biomarker for evaluating the prognosis of ACC patients.

Univariate analysis based on clinicopathological features showed that T classification (OS, *p* < 0.001; DFS, *p* < 0.001), distant metastasis (OS, *p* < 0.001; DFS, *p* < 0.001), nerve invasion (OS, *p* = 0.025; DFS, *p* = 0.029), and *SOX2* expression (OS, *p* < 0.001; DFS, *p* < 0.001) were significant risk factors affecting OS and DFS of 131 ACC patients (Table 2). To further investigate the prognostic values of *SOX2* in clinical outcomes in patients with ACC, we performed multivariate analyses using Cox proportional hazards regression model; criteria for model selection was indicated by univariate analysis with *p* < 0.05. The results revealed that distant metastasis (OS, *p* = 0.011; DFS, *p* = 0.021), and *SOX2* expression (OS, *p* = 0.035; DFS, *p* = 0.042) were two most important prognostic factors.

## 3. Discussion

In our study, we evaluated the expression of *SOX2* in ACC tissue using IHC, western blot, and qPCR analysis. We demonstrated that *SOX2* expression was associated with the development of ACC. Additionally, *SOX2* expression were significantly correlated with advanced T stage and distant metastasis, suggesting that *SOX2* expression might be of clinical relevance in the progression of ACC. Moreover, the univariate and multivariate analyses clearly demonstrated that *SOX2* expression was a statistically significant risk factor affecting OS and DFS of patients with ACC and were an independent prognostic marker for ACC patients.

The role of the *SOX* gene family in the cancerogenesis has been attributed to their properties involved in the regulation of cell differentiation, proliferation, and survival in multiple essential processes. All members of the *SOX* gene family share a non-canonical 79 amino acid DNA-binding domain known as the high mobility group (HMG) box domain [16]. In 2004, Koopman and collaborators published one of the first reviews on the involvement of the *SOX* gene family in cancer [24]. Graham *et al.*, reported that changes in *SOX4* gene expression may play a role in commitment to the differentiated phenotype in the normal and malignant mammary gland [25]. Pramoonjago *et al.* demonstrated that *SOX4* contributes to the malignant phenotype of ACC cells by promoting cell survival [26]. *SOX9* broadly plays a role in cancerogenesis and is overexpressed in many types of human cancers, where *SOX9* exhibits pro-oncogenic properties of promoting cell proliferation, inhibiting cell senescence, and collaborating with other oncogenes in neoplastic transformation [27]. *SOX10* expression in ACC appears to be a part of a highly coordinated transcriptional program characteristic of cancers with basal/myoepithelial features [28]. Moreover, TGF-beta induced expression of *SOX2* was mediated by *SOX4* in glioma-initiating cells [29]. To date, more and more findings support the involvement of different *SOX* genes in cancer development.

*SOX2* is a key regulator for maintaining the pluripotency and self-renewal of embryonic stem cells and contributes to the reprogramming of differentiated somatic cells back to a pluripotent stem cell state [30]. In particular, *SOX2* is a key factor conferring “stemness” characteristics and maintaining stem cell identity [31–33]. The stemness program can also have an important role in cancer because self-renewal is a hallmark for cancer-initiating cells/tumor-propagating cells. Other important roles of *SOX2* protein in cancer progression focused on its positive contribution to many physiological processes of cancer cells, such as proliferation and growth, cellular migration and invasion, maintenance of stemness and tumorigenicity, apoptosis and chemoresistance, metastasis and tumorigenesis [34]. For instance, in prostate cancer, *SOX2* promotes tumorigenesis and decreases apoptosis by activating the EGFR/PI3K/AKT pathway [35], and plays a critical role in EGFR-mediated self-renewal of human prostate cancer stem-like cells [36]. In lung cancer, *SOX2* is highly upregulated and it promotes cell migration and proliferation, acting as a lineage survival oncogene and driving cells toward squamous differentiation and pluripotency [37]; and *SOX2*, together with protein kinase Cι (PKCι), drive tumorigenesis by establishing a cell-autonomous hedgehog signaling axis [38]. Also of note, silencing of *SOX2* by brachyury knockdown inhibited epithelial-mesenchymal transition (EMT) in adenoid cystic carcinoma [39]. Recently, accumulating evidence demonstrated that *SOX2* expression level is closely correlated with clinical progression and poor prognosis among various tumor types, including hepatocellular carcinoma [40], colorectal cancer [41], lung cancer [42], gastric cancer [21], and laryngeal squamous cell carcinoma [43]. Our findings are consistent with several publications from other groups [44–46]. As increased *SOX2* expression is seen in tumor, but not adjacent normal tissue, we supposed that elevated *SOX2* expression may be related to ACC.

Recently, Yang *et al.* [42] reported that *SOX2* expression was associated with clinical stage and lymph node status in patients with small cell lung cancer. Tang *et al.* [43] suggested that *SOX2* expression was significantly associated with tumor T classification, clinical stage, lymph node metastasis, and recurrence in laryngeal squamous cell carcinoma. Du *et al.* [47] indicated that *SOX2* positive expression showed a significant association with large tumor size in tongue squamous cell carcinoma. Zhang *et al.* [21] reported that patients with strong *SOX2* expression showed deeper invasion and III–IV clinical stages compared to patients with low *SOX2* expression in gastric cancer. In subsequent exploration, we analyzed the correlation of *SOX2* expression with clinical features of ACC patients. We found that although *SOX2* protein expression was not associated with patient’s age, gender, histological type, and nerve invasion, it was positively correlated with T classification and distant metastasis. These results suggested *SOX2* is involved in the progression of ACC.

To date, there have been several studies describing the prognostic significance of *SOX2* expression in malignancies. In the present study, for the first time, we analyzed the correlation between *SOX2* expression and the survival rate of 131 ACC patients. The results revealed a close association between *SOX2* expression and clinical outcome. Our study also demonstrated that high expression of *SOX2* was one of the most important prognosis factors in the univariate and multivariate analysis. Therefore, we hypothesize that elevated *SOX2* expression can be used as a more reliable marker and it may represent a therapeutic target in ACC.

## 4. Experimental Section

### 4.1. Patients

Primary tumor specimens were obtained from 131 patients (75 and 56 males, ranging in age from 36 to 78 years) diagnosed with ACC who underwent radical surgery at the Department of Oral and Maxillofacial Surgery, China Medical University (Shenyang, China) between 2001 and 2007. Paired adjacent non-cancerous salivary gland tissues, located at least 1 cm away from the tumor, were collected from the surgically treated ACC patients. The pathology diagnosis of ACC was made on the basis of morphologic and IHC findings evaluated by two independent pathologists. Patients did not receive chemotherapy, radiotherapy or immunotherapy prior to surgery. Clinical and pathological characteristics including age, gender, tumor size, histological type, tumor node metastasis (TNM) classification, tumor grade, metastasis, and nerve invasion were summarized in Table 1. Tumor staging was assessed according to UICC 2002 staging system, and the histological types were classified according to the World Health Organization classification.

Informed consent was obtained from all ACC patients. The present study conformed to the ethical standards of the World Medical Association Declaration of Helsinki and was approved by the Ethics Committee of China Medical University ([2001] No. 6, China Medical University Ethics Committee).

### 4.2. Immunohistochemistry and Scoring

Sections were deparaffinized in xylene and rehydrated with graded alcohol. Antigen retrieval was performed using citrate buffer (pH 6.0) and sections were held in Tris buffered saline (TBS). Endogenous peroxidase activity was blocked by incubation in 3% hydrogen peroxide for 10 min. The slides were then incubated with monoclonal mouse anti-*SOX2* antibody (Sigma, St. Louis, MO, USA) at 1:100 dilution. Staining for anti-*SOX2* antibody was performed at 4 °C overnight. Then, the slides were incubated with horseradish peroxidase-conjugated rabbit anti-mouse IgG, and the color was developed with the DAB Horseradish Peroxidase Color Development Kit (Maixin Co., Fuzhou, China).

An immunoreactivity score system was applied as described previously [48]. The percentage of *SOX2*-positive cells was scored as 0 (<5%, negative); 1 (5%–25%, sporadic); 2 (25%–50%, focal); 3 (>50%, diffuse). The intensity of *SOX2*-positive staining was scored as 0 (negative); 1 (weak staining); 2 (moderate staining); and 3 (strong staining). Both the percent of positive cells and cell staining intensity were decided in a double-blinded manner. The total score was determined by the following formula: Staining score = intensity × positive rate. The sum of the percentage and intensity score was used as the final *SOX2* staining score and was defined as follows: 0–4, low expression, and 6–9, high expression.

### 4.3. Western Blotting Analysis

The ACC specimens were homogenized in a RIPA lysis buffer (Sigma-Aldrich, Steinheim, Germany) and 40 μg of total protein was separated through electrophoresis on a SDS-PAGE gel and transferred to polyvinylidene fluoride (PVDF) membranes (GE Healthcare, Barrington, IL, USA). The membrane was blocked at room temperature for 1 h in TBS containing 0.1% Tween-20 (TBST) and 5% fat-free powdered milk, and incubated overnight with primary antibodies (*SOX2* antibody and GAPDH antibody) at 4 °C, followed by horseradish peroxidase (HRP)-conjugated secondary antibody (1:2000 dilution) for 1 h at room temperature. Then, related protein was visualized by chemiluminescence detection (GE Healthcare, Barrington, IL, USA).

### 4.4. RNA Extraction, Reverse Transcription and Real-Time Quantitative PCR (RT-qPCR)

Real-time PCR was used to measure the expression of *SOX2* mRNA in 30 pairs of ACC cancer tissue samples and corresponding noncancerous tissue samples. Total RNA from cancer tissues were extracted using Trizol (Invitrogen, Carlsbad, CA, USA). The method for quantification and assessment of RNA samples is spectrophotometric measurement (NanoDrop, Thermo Scientific, FL, USA) at 260 and 280 nm, and *A*_260_/*A*_280_ ratios are very high in the 1.9–2.1 range. One microgram of RNA was used as a template for complementary DNA synthesis using Quantitect Reverse Transcription Kit (TaKaRa, Shiga, Japan). The SYBR green dye (Takara, Shiga, Japan) was used for the amplification of cDNA. The mRNA levels of *SOX2* and the internal standard β-actin were measured by real-time quantitative PCR in triplicate using an Mx3000P™ real-time PCR system by Agilent (Stratagene, La Jolla, CA, USA). The sequence for *SOX2* sense primer was 5′-AACAGCCCGGACCGCGTCAA-3′, and for antisense primer was 5′-TCGCAGCCGCTTAGCCTCGT-3′. The primers of β-actin mRNA were 5′-TGGCATCCACGAAACTAC-3′ for sense, and 5′-CTTGATCTTCATGGTGCTG-3′ for antisense. Relative gene expression was calculated with Mx3000P Software version 2.0 (Stratagene) by using the 2^−ΔΔ^*^C^*^q^ method. All steps in this manuscript were based on the MIQE guidelines for assessment of the RT-qPCR analyses [49,50].

The RT-qPCR amplification efficiency was determined from the slope of the standard curve generated with serial dilutions of ACC cDNA template for each gene. Ten-fold serial dilutions of the ACC cDNA standard, spanning 1 × 10^7^ to 1 × 10^3^ copies/μL, were used and tested in triplicate. Standard curve was plotted as the mean *C*q values versus the log cDNA copy numbers. Regression analysis, standard curve slopes and amplification efficiencies were calculated using automated software (Mx3000P Software version 2.0, Stratagene). To establish β-actin cDNA amplification equally in different tissues, cDNA standards in different ACC tissue and non-cancerous tissues were used in PCR reaction system. The method of amplification efficiency was as above.

### 4.5. Statistical Analysis

All analyses were performed using SPSS 17.0 statistical package (SPSS, Inc., Chicago, IL, USA). The Chi-square tests were used to analyze the relationship between the *SOX2* expression levels and various clinicopathological features. Survival time was calculated from the date of ACC diagnosis to the date of death or last follow-up. The effect of *SOX2* expression on the overall survival (OS) and disease-free survival (DFS) was evaluated by Kaplan–Meier method and log-rank test. The Cox proportional-hazard analysis was used for univariate and multivariate analyses to explore the effect of the clinicopathological variables and *SOX2* expression on survival. In all analyses, a probability (*p*) value of less than 0.05 was considered to indicate significance.

## 5. Conclusions

Taken together, our results provide evidence that *SOX2* expression may be involved in the clinical progression and poor prognosis of ACC patients. However, it could serve as a potential independent prognostic factor for ACC patients. Although further studies are needed to clarify the role and mechanism of high *SOX2* expression in the progression of ACC, the present study provides new insights into the progression of ACC.

## Supplementary Information



## Figures and Tables

**Table 1.** Clinical characteristics according to *SOX2* expression in ACC.

Clinicopathological variables	Number of patients (%)	High *SOX2* expression	Low *SOX2* expression	*p* value
**Age**				
<50 years	46	25	21	0.186
≥50 years	85	57	28	

**Gender**				
Male	75	44	31	0.362
Female	56	38	18	

**Histological type**				
Cribriform/Tubular	102	61	41	0.278
Solid	29	21	8	

**T classification**				
I or II	77	40	37	0.003
III or IV	54	42	12	

**Distant metastasis**				
No	88	47	41	0.002
Yes	43	35	8	

**Nerve invasion**				
No	36	19	17	0.163
Yes	95	63	32	

**Table 2.** Univariable and multivariable analysis of ACC survival using Cox’s proportional hazards model.

Group	Univariable analysis			Multivariable analysis		
	
	HR	95% CI	*p*	HR	95% CI	*p*
**OS**						
Age (<50 *vs.* ≥50 years)	1.21	0.66–2.10	0.514	Not included in model		
Gender (Male *vs.* Female)	1.38	0.89–2.33	0.440	Not included in model		
Histotological type (Cribriform/Tubular *vs.* solid)	1.29	1.17–2.99	0.509	Not included in model		
T classification (I,II *vs.* III,IV)	5.02	2.19–9.41	<0.001	2.11	1.07–4.26	0.264
Distant metastasis (Yes *vs.* No)	8.05	4.27–13.60	<0.001	5.49	2.91–9.99	0.011
Nerve invasion (Yes *vs.* No)	2.31	1.38–5.23	0.025	1.35	1.01–4.33	0.123
*SOX2* expression (High *vs.* Low)	4.71	2.08–8.63	<0.001	2.65	1.21–5.20	0.035

**DFS**						
Age (<50 *vs.* ≥50 years)	1.09	1.00–1.82	0.711	Not included in model		
Gender (Male *vs.* Female)	1.31	0.91–2.00	0.489	Not included in model		
Histotological type (Cribriform/Tubular *vs.* solid)	1.30	1.41–3.20	0.222	Not included in model		
T classification (I,II *vs.* III,IV)	4.73	2.02–8.25	<0.001	1.85	1.10–4.31	0.287
Distant metastasis (Yes *vs.* No)	7.81	3.44–12.50	<0.001	5.32	2.31–9.05	0.021
Nerve invasion (Yes *vs.* No)	2.22	1.09–4.82	0.029	1.34	1.00–3.40	0.199
*SOX2* expression (High *vs.* Low)	4.55	1.99–8.35	<0.001	2.57	1.44–5.07	0.042

OS, overall survival; DFS, disease-free survival; HR, hazard ratio.
