# Supplementary material for: High Expression of SOX2 Is Associated with Poor Prognosis in Patients with Salivary Gland Adenoid Cystic Carcinoma
Source: Int J Mol Sci. 2014 May 13;15(5):8393–406. doi: 10.3390/ijms15058393 (PMC4057738; doi:10.3390/ijms15058393)
Supplement: Supplementary file 1 [file ijms-15-08393-s001.pdf]

Supplementary Information

Figure S1. The standard curves and amplification plots of *SOX2* and β-actin. (**a**) Standard curve of *SOX2*; (**b**) Amplification plots of SOX2; (**c**) Standard Curve of β-actin; (**d**) Amplification plots of β-actin; (**e**,**f**) Standard curves of β-actin in ACC tissue and non-cancerous tissue.

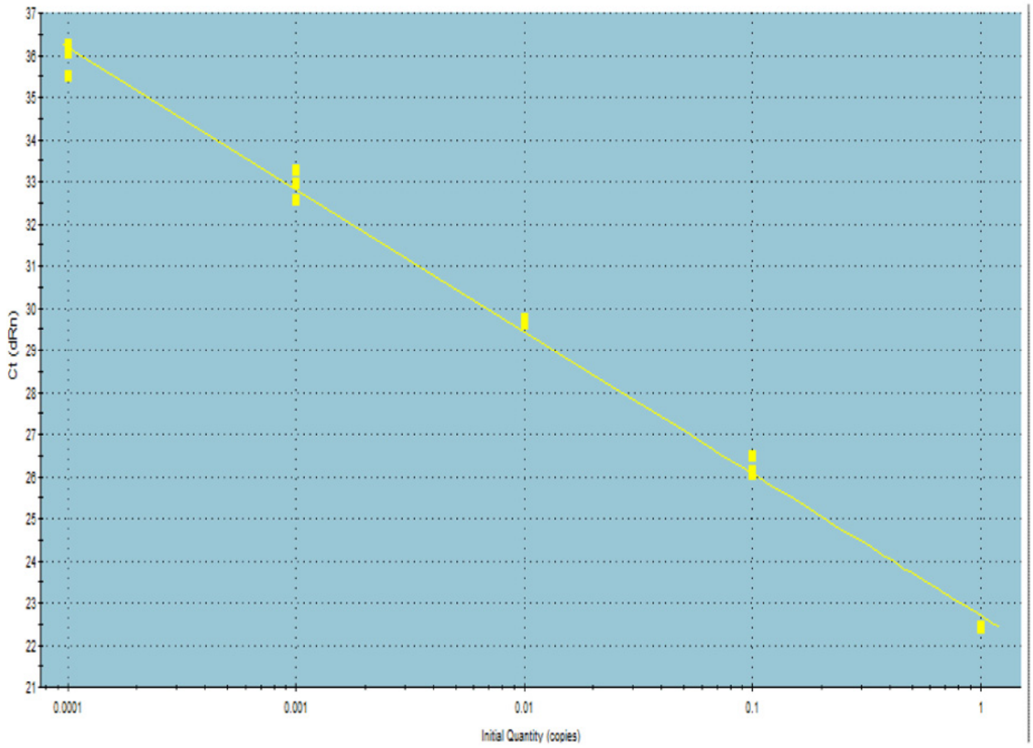

(**a**)

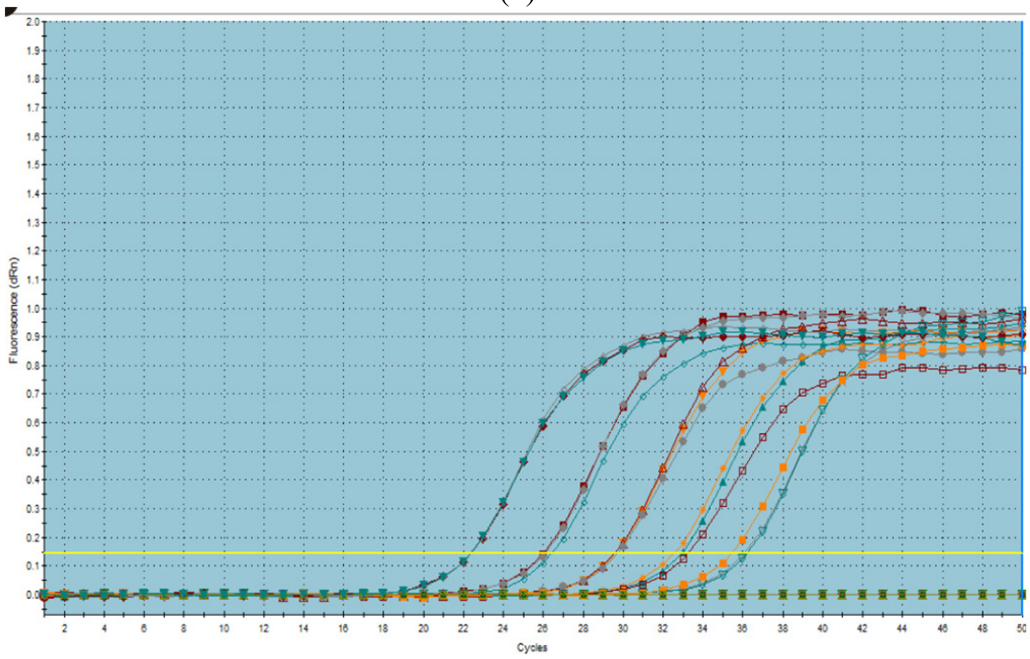

(**b**)

Figure S1. *Cont.*

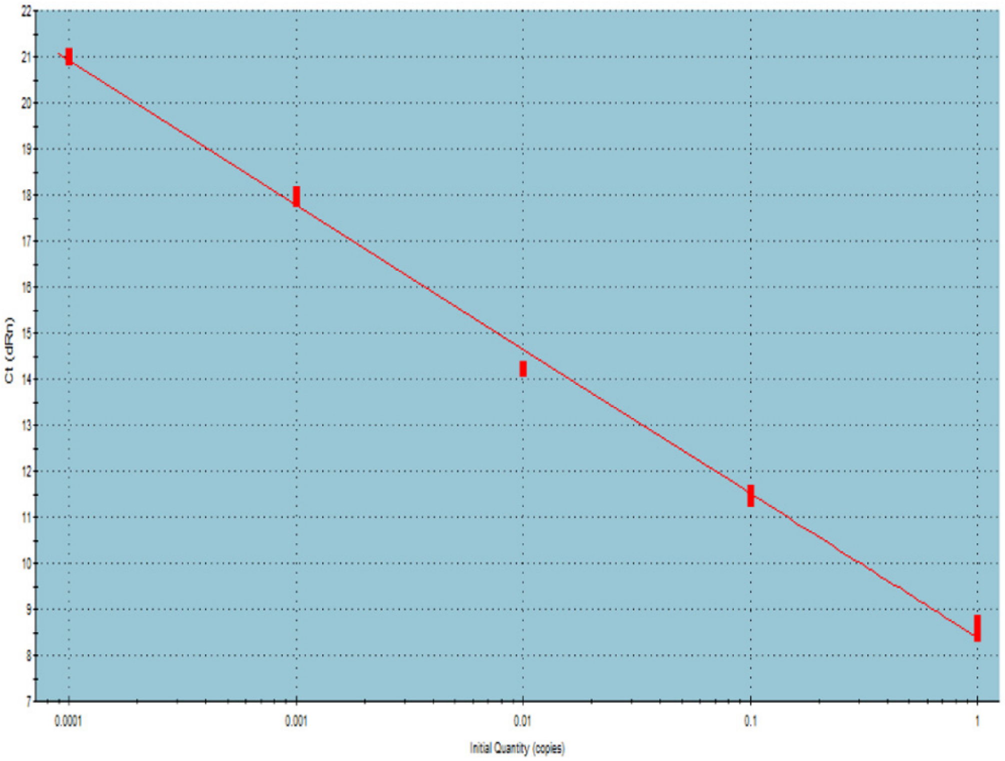

(**c**)

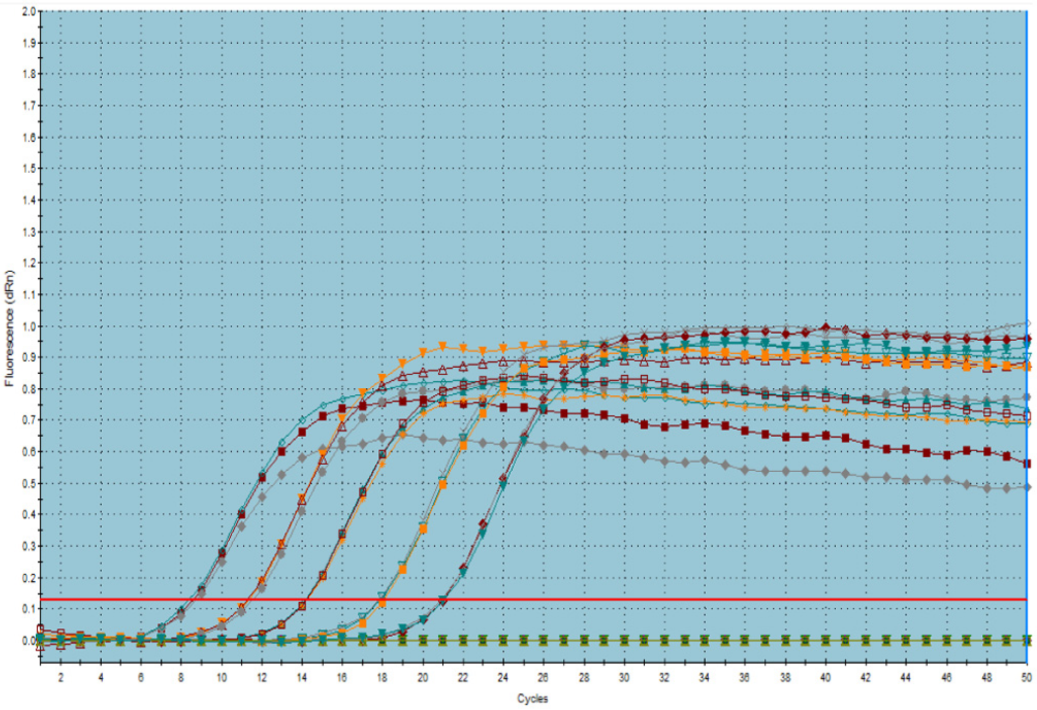

(**d**)

Figure S1. *Cont.*

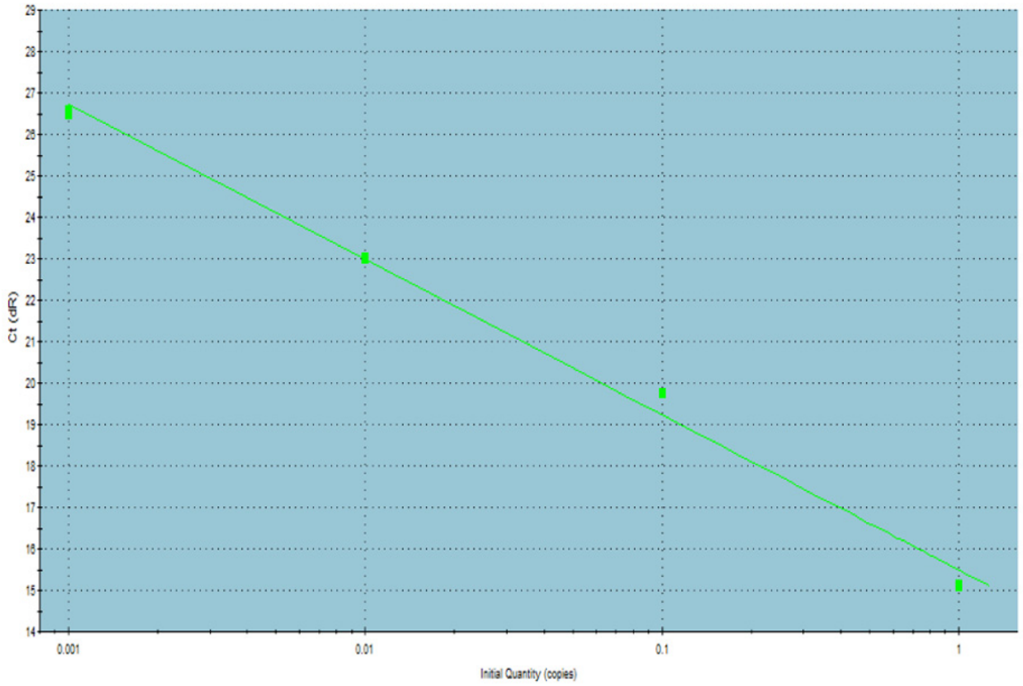

(e)

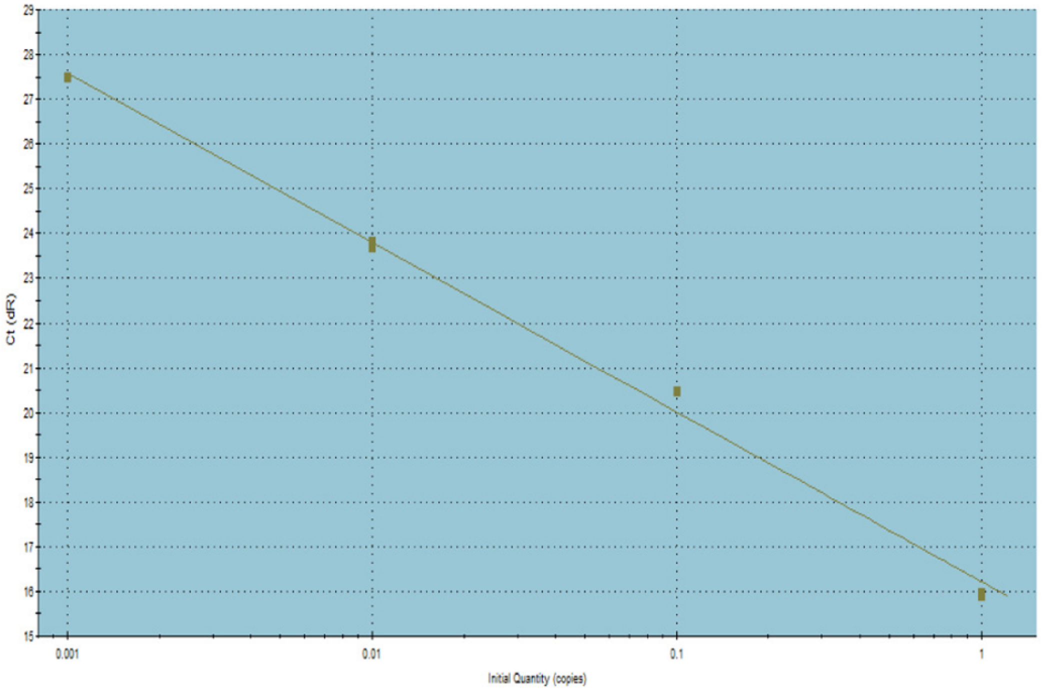

(f)

© 2014 by the authors; licensee MDPI, Basel, Switzerland. This article is an open access article distributed under the terms and conditions of the Creative Commons Attribution license (http://creativecommons.org/licenses/by/3.0/).
